# ASO Author Reflections: Optimizing Blood Sample Collection in Randomized Controlled Trials: Lessons from PREOPANC-2

**DOI:** 10.1245/s10434-025-16981-y

**Published:** 2025-02-12

**Authors:** Esther N. Dekker, Dana A. M. Mustafa, Bas Groot Koerkamp

**Affiliations:** 1 Department of Surgery, Erasmus MC Cancer Institute, Rotterdam, The Netherlands; 2 Department of Pathology, Erasmus MC, Tumor Immuno-Pathology Laboratory, Rotterdam, The Netherlands

## Past

Randomized controlled trials (RCTs) are crucial for validating predictive biomarkers in cancer research. However, the collection of blood samples in multicenter RCTs poses significant challenges. Our study addressed the key question: Is it feasible to collect blood samples with high yield in a nationwide, investigator-initiated RCT for pancreatic cancer?

## Present

The PREOPANC-2 trial demonstrated that high-yield blood sample collection is achievable in a multicenter RCT setting. We successfully collected 87% of eligible blood samples from 363 patients across 19 centers in the Netherlands. This high yield was particularly notable for pretreatment blood samples, with a 96% collection rate. Our findings highlight the importance of meticulous planning, standardized protocols, and dedicated research teams in each participating center. The study also identified potential areas for improvement, such as scheduling failures and logistical barriers, including challenges with blood sample transportation.^1^

## Future

Future RCTs should prioritize blood sample collection as an integral part of study design. Funding agencies should require and fund blood sample collection and storage for clinical trials in oncology. Researchers need to develop robust strategies to maintain high collection rates throughout the trial duration, especially during long-term follow-up. Standardization of blood collection protocols across different trials could facilitate biomarker validation and discovery. Additionally, there is a need for guidelines on the ethical use and storage of collected samples to ensure their optimal utilization in future research.^2^As personalized medicine advances, the integration of blood-based biomarkers into clinical decision-making will become increasingly important, underscoring the need for high-quality biospecimen collection in RCTs.^3^
